# Investigation of major amino acid residues of anti-norfloxacin monoclonal antibodies responsible for binding with fluoroquinolones

**DOI:** 10.1038/s41598-021-96466-6

**Published:** 2021-08-25

**Authors:** Patamalai Boonserm, Songchan Puthong, Thanaporn Wichai, Sajee Noitang, Pongsak Khunrae, Sarintip Sooksai, Kittinan Komolpis

**Affiliations:** 1 Program in Biotechnology, Faculty of Science, Chulalongkorn University, Bangkok, Thailand; 2 Institute of Biotechnology and Genetic Engineering, Chulalongkorn University, Bangkok, Thailand; 3 King Mongkut’s University of Technology Thonburi, Bangkok, Thailand; 4 Food Risk Hub, Research Unit of Chulalongkorn University, Bangkok, Thailand

**Keywords:** Biotechnology, Computational biology and bioinformatics

## Abstract

It is important to understand the amino acid residues that govern the properties of the binding between antibodies and ligands. We studied the binding of two anti-norfloxacins, anti-nor 132 and anti-nor 155, and the fluoroquinolones norfloxacin, enrofloxacin, ciprofloxacin, and ofloxacin. Binding cross-reactivities tested by an indirect competitive enzyme-linked immunosorbent assay indicated that anti-nor 132 (22–100%) had a broader range of cross-reactivity than anti-nor 155 (62–100%). These cross-reactivities correlated with variations in the numbers of interacting amino acid residues and their positions. Molecular docking was employed to investigate the molecular interactions between the fluoroquinolones and the monoclonal antibodies. Homology models of the heavy chain and light chain variable regions of each mAb 3D structure were docked with the fluoroquinolones targeting the crucial part of the complementarity-determining regions. The fluoroquinolone binding site of anti-nor 155 was a region of the HCDR3 and LCDR3 loops in which hydrogen bonds were formed with TYR (H:35), ASN (H:101), LYS (H:106), ASN (L:92), and ASN (L:93). These regions were further away in anti-nor 132 and could not contact the fluoroquinolones. Another binding region consisting of HIS (L:38) and ASP (H:100) was found for norfloxacin, enrofloxacin, and ciprofloxacin, whereas only ASP (H:100) was found for ofloxacin.

## Introduction

An antibody is an immunoglobulin protein that binds to specific antigens. Each antibody consists of four polypeptides—two heavy chains (H) and two light chains (L) joined to form a “Y”-shaped molecule. The amino acid sequence in the tips of the “Y” varies greatly among different antibodies. This variable region gives the antibody its specificity for a specific antigen^1,2^. The variable region is further subdivided into hypervariable and framework regions^3^. The hypervariable regions have a high number of different amino acids that make up the antigen-binding site^4^. A change in the amino acid sequence in this region can greatly affect the specificity and affinity of the binding between the antibody and antigens.

Antibodies can be either polyclonal, secreted by different B cell lineages, or monoclonal, secreted by a single B cell lineage. Both polyclonal and monoclonal antibodies (pAbs and mAbs, respectively) have their advantages and disadvantages, which make them useful for different applications. However, mAbs are often more useful than pAbs, because mAbs can be produced without limit by the cultivation of immortal hybridoma cell lines^5^. The properties of mAbs are also more consistent than those of pAbs in batch-to-batch production. To obtain mAbs of two antigens with closely related structures, individual hybridoma cell lines must be generated. The development of such hybridoma cell lines is time-consuming, and the binding abilities of the obtained mAb might be relatively poor. It may be possible to overcome these problems by modifying the variable region of an existing mAb, which can bind to an antigen with a similar structure. Information about the amino acid sequences that govern the binding ability of antibodies is essential for such modification to be undertaken^6^. Molecular docking has been shown to be a powerful tool for drug discovery and protein binding studies. Currently, there are several molecular docking applications and servers available, including both commercial and free formats based on different algorithms^7^, including AutoDock^8^, SwissDock^9^, GOLD (Genetic Optimization for Ligand Docking)^10^, HADDOCK^11^, and MVD^12^. These programs can predict the ligand conformation and position within the targeted protein’s binding site based on the structure of the molecules involved. In antibody-antigen docking study, computational docking has been used to design antibodies with improved binding properties. For example, Shaun et al.^13^ generated higher affinity variants for three antibody targets by computationally selecting mutations that improved antibody–antigen interaction energy. Similary, Poosarla et al.^14^ developed a computational framework for the de novo design of fully human antibody variable domains to bind any specified antigen by assembling the six best-scored modular antibody parts. In addition, a fundamental characteristic of the immune system is its ability to generate novel protein recognition sites continuously, Ab–Ag interaction. For example, Keskin^15^ used X-ray crystallographic structures of Ab–Ag complexes to explain principles of the molecular protein–protein interaction. Moreover, Sheng et al.^16^ used the molecular docking software AutoDock Vina (http://vina.scripps.edu/index.html) to show the interaction of the cyclic peptide inhibitor with both SARS-CoV-2 M^pro^ and the highly homologous SARS-CoV-2 M^pro^.

Fluoroquinolones (FQs) are a group of antibiotics used against gram-negative and gram-positive bacteria in fish, livestock, and poultry. These antibiotics are valuable because they inhibit DNA gyrase and topoisomerase and other enzymes essential for bacterial DNA replication^17^. However, the use of antibiotics in large quantities and over long periods of time can lead to the accumulation of antibiotic residues in animal products such as meat and milk, which are subsequently used for human consumption. Unintentional ingestion of such residue-contaminated products could cause health issues to humans, such as the development of antimicrobial resistance and allergies^18^. Although drug residue surveillance is practiced and maximum residue limits are imposed in many countries^19^, there is still a need to develop simple, responsive, specific, and inexpensive methods for the detection of antibiotic residues. Detection based on immunological methods such as enzyme-linked immunosorbent assay (ELISA) and lateral flow immunoassays has been widely used for screening in food safety applications^20,21^. In these methods, either an antibody with broad specificity to FQs or several specific antibodies for each antibiotic are required.

Our research group has produced mAbs against norfloxacin. Among the mAbs obtained, anti-nor 132 and anti-nor 155 showed high sensitivity, with different specificities. In this research, the bindings of the two mAbs with the FQs norfloxacin, enrofloxacin, ciprofloxacin, and ofloxacin were analyzed using the AutoDock Vina program^22^ and the possible major sites or sequences that govern the binding characteristics of the mAbs are identified. This knowledge is essential to design amino acid sequence modifications, which will produce desired properties of the mAbs.

## Results

### Binding ability of anti-nor 132 and anti-nor 155

**Figure 1:**
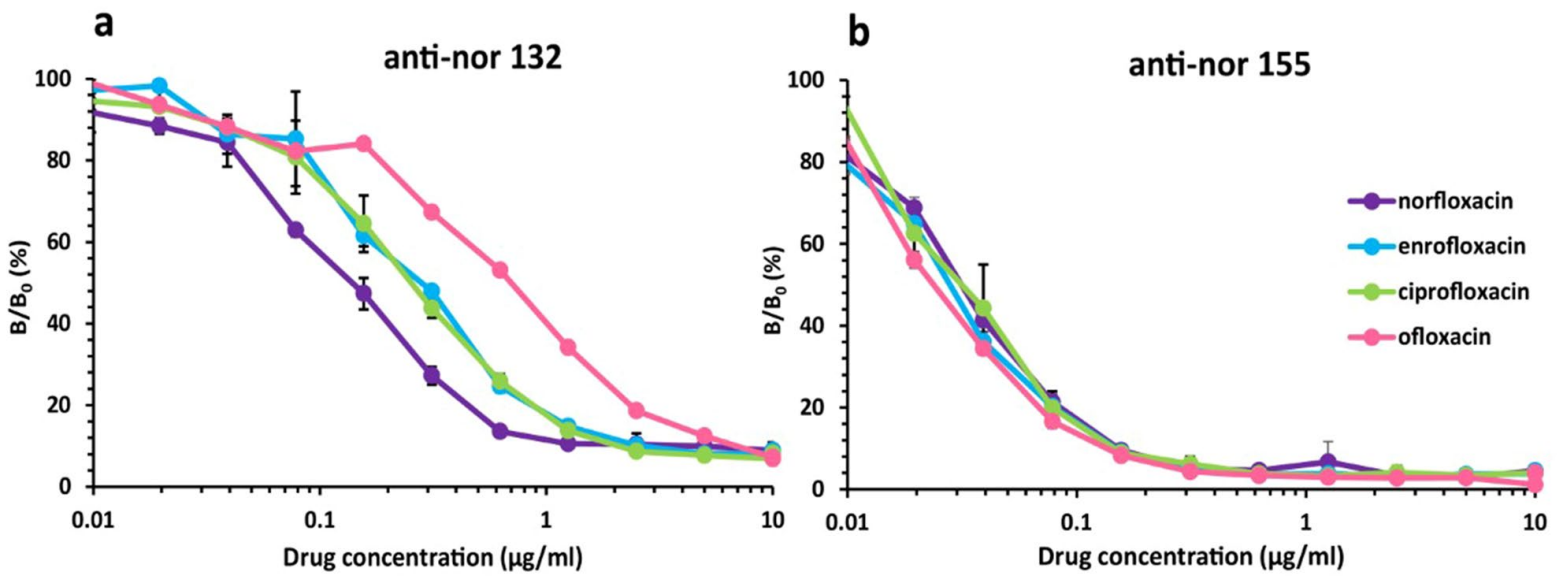
Dose–response curves of the monoclonal antibody. (**a**) anti-nor 132 and (**b**) anti-nor 155 to norfloxacin (), enrofloxacin (), ciprofloxacin () and ofloxacin () analyzed by an indirect competitive ELISA using anti-nor (100 µL/well) and different concentrations of FQs of competitors (50 µL/well), adding goat anti-mouse IgG-horse radish peroxidase diluted at 1:10,000. B/B_0_ represents the ratio of the ELISA mean absorbance value of the antibody binding response in the presence of free antigen to that in the absence of antigen.

Indirect competitive ELISA was used to study the binding of the mAbs and FQs such as norfloxacin, enrofloxacin, ciprofloxacin, and ofloxacin. It could be seen from the dose–response curves (Fig. 1) that the absorbance values decreased with respect to the concentration of all competitors.

These results indicated that both mAbs could bind to all tested FQs. The dose–response curves of anti-nor 155 to all tested FQs were not distinctly different whereas those of anti-nor 132 were relatively different. This observation suggested that anti-nor 155 bound to the tested FQs at approximately the same level, whereas the binding levels of anti-nor 132 were moderately different. The percentages of cross-reactivity calculated from the ratio of the 50% inhibition concentration (IC_50_) values of anti-nor 155 to all tested FQs were in the range of 62–89%, whereas those of anti-nor 132 were in the range of 22–61%, as compared with that of norfloxacin (100%) (Table 1). On the basis of the calculated limit of detection (LOD) values, the sensitivity of anti-nor 132 to all tested FQs was higher than that of anti-nor 155.

**Table 1:** Detection sensitivity and binding specificity of anti-nor 132 and anti-nor 155.

Competitors	LOD (µg/mL)		IC_50_ (µg/mL)		Cross-reactivity (%)	
	Anti-nor 132	Anti-nor 155	Anti-nor 132	Anti-nor 155	Anti-nor 132	Anti-nor 155
Norfloxacin	0.009	0.017	0.139	0.051	100	100
Enrofloxacin	0.006	0.023	0.226	0.057	61	89
Ciprofloxacin	0.011	0.034	0.258	0.083	54	62
Ofloxacin	0.005	0.060	0.637	0.063	22	81

Remarks: *LOD* Limit of detection, *IC*_*50*_ 50% Inhibition concentration.

### Amino acid sequence analysis and 3D structure prediction of the mAbs

**Figure 2:**
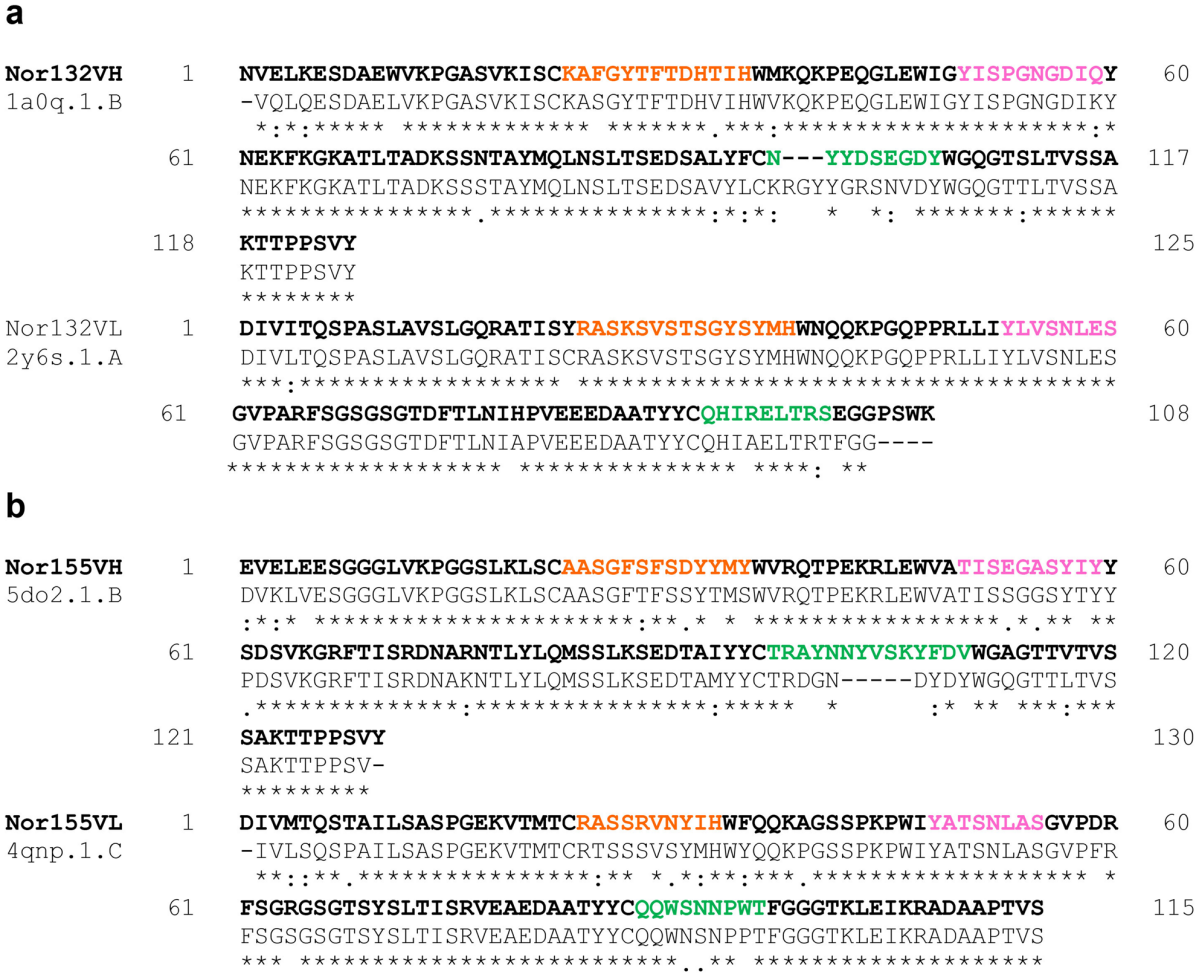
Sequence alignment of VH and VL. (**a**) Anti-nor 132 (VH:1a0q.1.B, VL:2y6s.1.A) and (**b**) anti-nor 155 (VH:5do2.1.B, VL:4qnp.1.C). Complementarity determining regions (CDRs) play the crucial part of variable regions, which are the high diversity of antigen specificities; CDR1, CDR2, and CDR3 are colored in orange, pink, and green, respectively. The amino acid sequences of the VH and VL regions of the mAbs and the template were aligned using the CLUSTALW server (www.genome.jp/tools-bin/clustalw).

The amino acid sequences of the heavy chain (VH) and light chain (VL) variable regions of anti-nor 132 and anti-nor 155 were submitted to GenBank, with the accession numbers MW452862, MW452863 and KJ623260.1, KR261578.1. The sequences were then submitted to the SWISS-MODEL automated protein modeling server. The molecular structure template of V_H_ and V_L_ of the mAbs were chosen from the highest sequence identities from the PDB Data Bank. The amino acid sequence of anti-nor 132 was found to match with the template of the heavy chain (VH) (PDB ID: 1a0q.1.B) and light chain variables (VL) (PDB ID: 2y6s.1.A) with similarities of 83.87% and 87.72%, respectively. In comparison, anti-nor 155 was found to match with the template of VH (PDB ID: 5do2.1.B) and VL (PDB ID: 4qnp.1.C) with 87.10% and 94.23% sequence identity, respectively (Fig. 2). It is possible that the difference in the amino acid chain length of the CDR of the template and the tested sequences could create a limitation and variation of the modelling outcome.

To evaluate whether the model was reliable and accurate, the Global Model Quality Estimation (GMQE) and Qualitative Model Energy Analysis (QMEAN) scores were calculated (Table 2). The GMQE provided the expected accuracy of a model built between the amino acid sequence of interest and the template. A score of 1 indicates 100% reliability. The QMEAN score uses the statistical potentials of mean force to provide global and local absolute quality estimates. A score close to zero indicates good agreement between the model structure and the target structure of similar size^23^. The obtained GMQE and QMEAN values in this study were in the reliable range^24^.

**Table 2:** Scores of the mAbs structure prediction by SWISS-MODEL.

mAb	Fragment	Template	Organism	GMQE	QMEAN
Anti-nor 132	VH	1a0q.1.B	*Mus musculus*	0.97	0.39
	VL	2y6s.1.A	*Mus musculus*	0.96	− 1.81
Anti-nor 155	VH	5do2.1.B	*Mus musculus*	0.84	0.02
	VL	4qnp.1.C	*Mus musculus*	0.96	− 0.6

### Molecular docking

Molecular docking of the target FQs and the mAbs was done by placing the ligand into the active site of the mAbs using AutoDock Vina, which uses a global optimizer to produce docking results for ligands with approximately 20 flexible bonds. The blind docking method was used^22,25^, because the binding pockets of the mAbs were unknown. Therefore, the whole molecule of the mAb was enclosed into a grid box. The FQ poses were focused on the subdomains HCDR1, HCDR2, HCDR3, LCDR1, LCDR2, and LCDR3 of variable regions, which are favorable for interaction with the targeted FQs. The apposition of complementary shapes results in numerous contacts between the amino acids at the binding surfaces and the FQs. A combination of hydrogen bonds, electrostatic interactions, van der Waals interactions, and hydrophobic interactions affects both the binding specificity and the binding strength^26,27^. The best-fitted binding position was justified on the basis of the least negative-sum component of all relevant energies. The Gibbs free energy of binding (ΔG) between anti-nor 132 and all FQs was in the range − 7.3 to − 7.8 kcal/mol, whereas that of the binding between anti-nor 155 and all FQs was in the range − 7.2 to − 7.5 kcal/mol. These values indicated a stable binding between the mAbs and the FQs. However, these docking free energies were not real free energies. Instead, they were used to evaluate which of the conformations best complements the protein binding site^28^.

**Figure 3:**
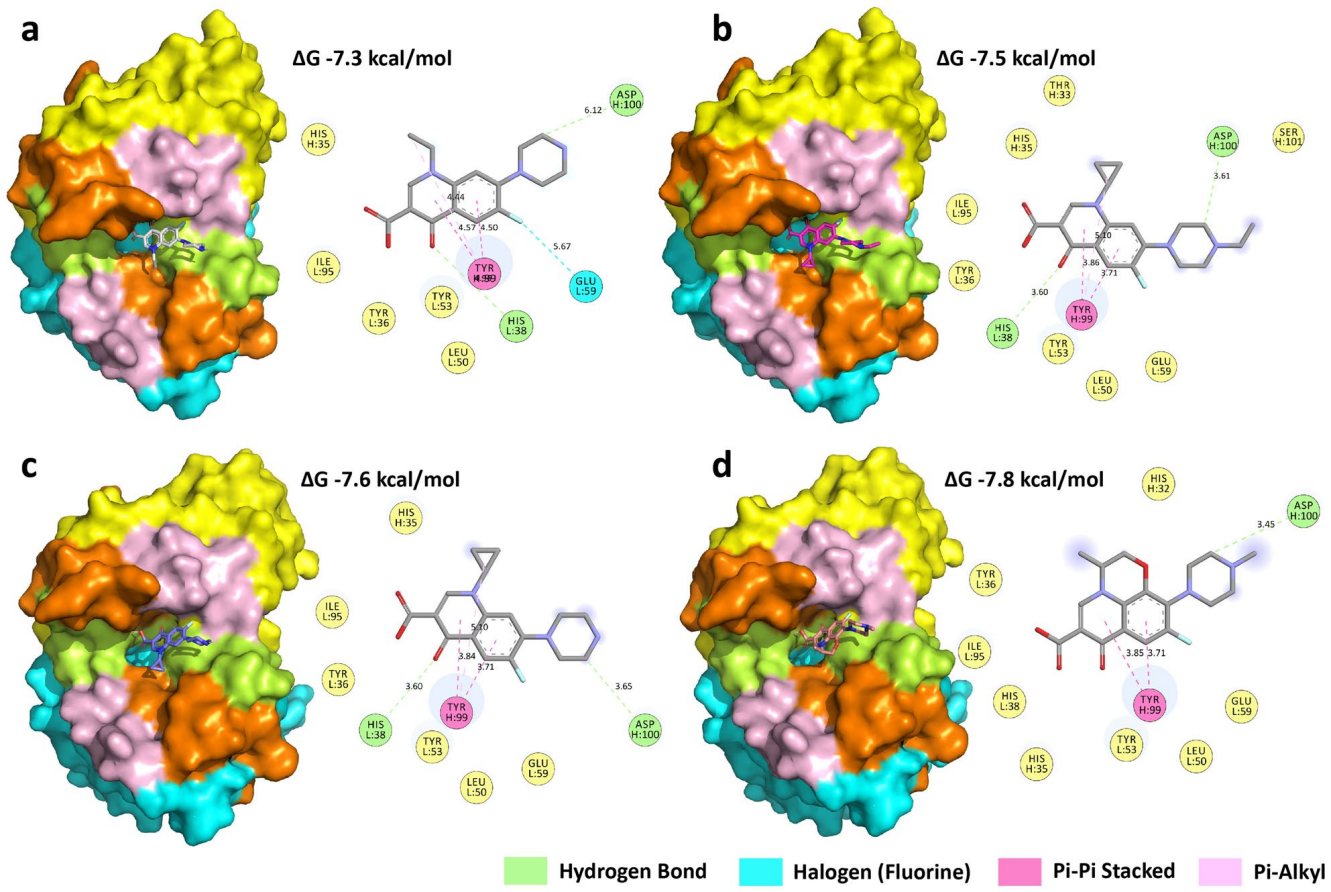
Schematic diagram of the binding of anti-nor 132 and target FQ molecules: (**a**) norfloxacin, (**b**) enrofloxacin, (**c**) ciprofloxacin, and (**d**) ofloxacin. The heavy (VH) and light (VL) chain variable regions of anti-nor 132 are shown in cyan and yellow, respectively. Complementarity determining regions (CDRs) are shown CDR1 (orange), CDR2 (light pink), and CDR3 (lemon). The 3D structures of the ligands norfloxacin (CID: 4539), enrofloxacin (CID: 71,188), ciprofloxacin (CID: 2764), and ofloxacin (CID: 4583) were obtained from the PubChem web site (https://pubchem.ncbi.nlm.nih.gov/). The docking images were generated using PyMOL Stereo 3D Quad-buffer (licensed version 2.5.1, Schrödinger Inc, USA) and the 2D images were generated using Discovery Studio 2019 Client (free version, BIOVIA Inc, China).

The best fitted models were exported and analyzed using Discovery Studio 2019 and PyMOL Stereo 3D Quad-buffer, respectively. The program predicted that the drugs docked with the CDR region of the mAbs very well, but at different positions. The docked conformation of anti-nor 132 with all ligands posed near the HCDR3 and LCDR1 loops. The pyrazine ring substituent of the FQs formed hydrogen bonds with the aspartic acid at position 100 of the heavy chain (ASP (H:100)) with an approximate distance of 3.64 Å on the HCDR3 region, presenting the β turn of anti-nor 132. The hydroxyl moiety of the ligands exposes another residue, histidine, at position 38 of the light chain (HIS (L:38)), with an approximate distance of 3.60 Å on the LCDR1 region, by electrostatic interactions. However, only ASP (H:100) at 3.45 Å was involved in binding with ofloxacin (Fig. 3).

**Figure 4:**
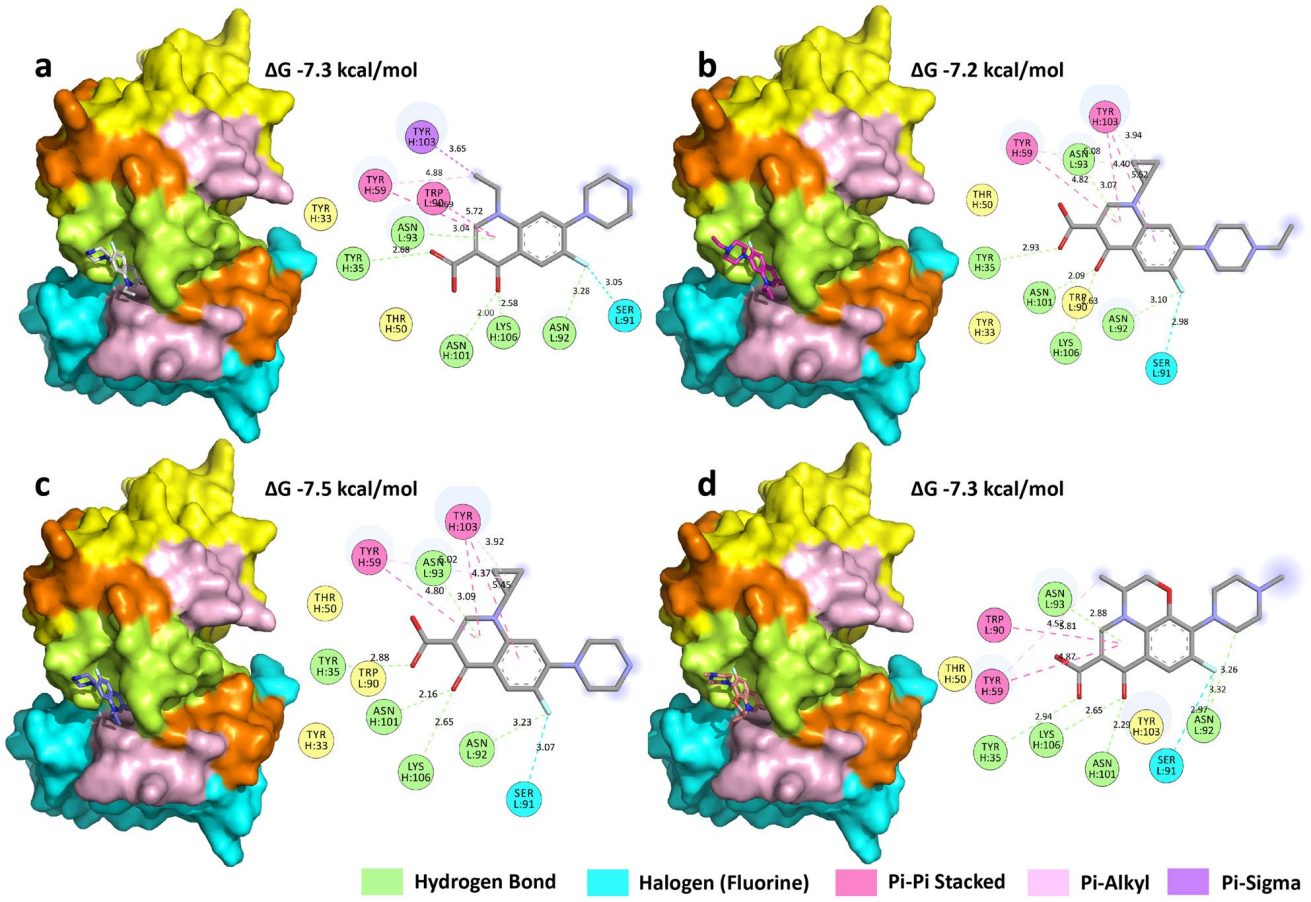
Schematic diagram of the binding between anti-nor 155 and target FQ molecules: (**a**) norfloxacin, (**b**) enrofloxacin, (**c**) ciprofloxacin, and (**d**) ofloxacin. The heavy (VH) and light (VL) chain variable regions of anti-nor 155 are shown in cyan and yellow, respectively. Complementarity determining regions (CDRs) are shown CDR1 (orange), CDR2 (light pink), and CDR3 (lemon). The 3D structures of the ligands norfloxacin (CID: 4539), enrofloxacin (CID: 71188), ciprofloxacin (CID: 2764), and ofloxacin (CID: 4583) were obtained from the PubChem web site (https://pubchem.ncbi.nlm.nih.gov/). The docking images were generated using PyMOL Stereo 3D Quad-buffer (licensed version 2.5.1, Schrödinger Inc, USA) and the 2D images were generated using Discovery Studio 2019 Client (free version, BIOVIA Inc, China).

In the docking conformation of the anti-nor 155–ligands complex, all ligands were positioned in the pocket site between the HCDR3 and LCDR3 regions (Fig. 4). These had a better fit at the anti-nor 155 pocket site because several amino acid residues were involved in forming hydrogen bonds with ASN (H:101), LYS (H:106), ASN (L:92), and ASN (L:93) on the HCDR3 and TYR (H:35) on the HCDR1 region, with an approximate distance of 2.14, 2.63, 3.22, 3.02, and 2.86 Å, respectively. Hydrophobic contributors such as TYR (H:59), TYR (H:103), and TRP (L:90) could also form π–π stacking interactions on the β turn, with the tested FQs. Hydrophobic contributors such as Tyr (H:59), Tyr (H:103), and Trp (L:90) could also form π–π stacking interactions on the β turn, with the tested FQs. Furthermore, fluorine could form halogen bond with Glu (L:59) of anti nor132 (LCDR2) and Ser (L:91) of anti nor155 (LCDR3) which strong binding like hydrogen bond.

## Discussion

Anti-nor 132 and anti-nor 155 were prepared using a conventional cell hybridization method. Although both mAbs came from the same fusion of splenocytes and myeloma cells, they possess different sensitivities and cross-reactivities. Anti-nor 132 had a broader range of the cross-reactivity values (22–100%) than anti-nor 155 (62–100%). It is possible that anti-nor 155 was less susceptible to the change in the moiety at the N1 position of the FQs than the anti-nor 132. In addition, both moieties at the N1 position of the core structure and the N position of the piperazine ring were important for the recognition of the mAbs, thus resulting in the differences in the cross-reactivity. However, it was not clear which moiety was more dominant than the other. The differences in cross-reactivity could also be due to differences in the amino acid sequence of the variable region of the mAb produced from different monoclones. The numbers and types of amino acids that were predicted to form bonds to the FQs were also different. Molecular docking simulation study suggested that ASP (H:100) and HIS (L:38) were important to the binding of anti-nor 132, and three FQs, norfloxacin, enrofloxacin, and ciprofloxacin, whereas only ASP (H:100) was found to be involved in binding with ofloxacin. The pyrazine ring carbon substitute on one side of the FQ structure binds to aspartic acid, because its side chain has a carboxylic acid group, which can bind and form hydrogen bonds. On the other side of the FQs, the hydroxyl substitute binds to histidine by charge–charge interaction. The phenyl group of the FQs investigated in this study could form π–π stacking, the weak interactions play when aromatic rings are stacked parallel to one another, with an aromatic ring of Tyr (H:99) residues. In the case of anti-nor 155, five amino acid residues TYR (H:35), ASN (H:101), LYS (H:106), ASN (L:92), and ASN (L:93) were predicted to govern the binding to all tested FQs. The hydroxyl group of TYR can form a hydrogen bond with the carboxyl group of the ligand^29^. ASN is an amino acid containing an amide group that can accept and donate hydrogen bonds and can therefore bind with hydroxyl substitutes through electrostatic interactions^30^. In the case of LYS, a positively charged amine side chain can bind with the negatively charged hydroxyl substitute of ligands^31^.

However, in this study, the docking study was performed without the effect of solvent on the mAb structures to reduce the variations. The solvent factor could have influence on binding and on selecting which amino acids are crucial for binding. A solvated potential model that approximates the potential energy of a solvated protein by projecting the solvent information into the protein structure has been proposed^32^. In addition, a multi-objective evolutionary algorithm has also been proposed to include the effect of solvent in predicting the three-dimensional structure of a protein to improve accuracy and efficiency of the prediction^33^.

**Figure 5:**
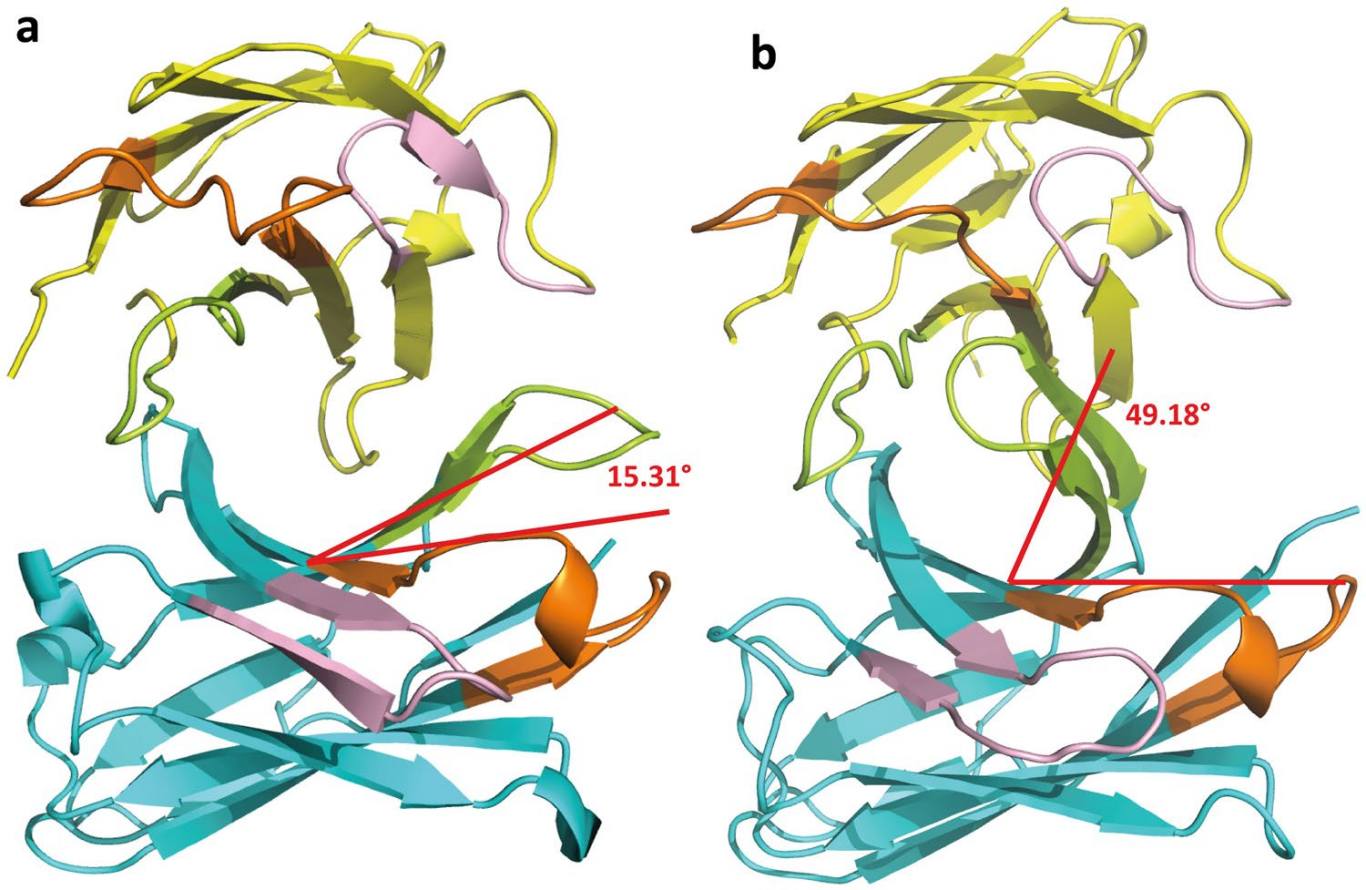
Conformation snapshots. (**a**) Anti-nor 132 and (**b**) anti-nor 155. The angles between the beta-sheets of the CDR region are represented by red lines, generated in visual molecular dynamics (VMD) version 1.9.4. The heavy (VH) and light (VL) chain variable regions of mAbs are shown in cyan and yellow, respectively. Complementarity determining regions (CDRs) are shown CDR1 (orange), CDR2 (light pink), and CDR3 (lemon). The 3D structures were generated using PyMOL Stereo 3D Quad-buffer (licensed version 2.5.1, Schrödinger Inc, USA).

The docking study indicated that the FQ-binding areas of anti-nor 155 were the HCDR3 and LCDR3 loops, which form a binding pocket site, resulting in a tight interaction. Because all tested FQs could interact with those five amino acid residues and be present in the binding pocket, the cross-reactivities between anti-nor 155 and the FQs tested were different, but they were in the same range. In the case of anti-nor 132, the binding loops found in anti-nor155 moved further away until they were unable to make a good contact with the FQs in order to form an appropriate binding pocket site (Fig. 5). The binding occurred at different areas, depending on the position of the amino acid that could interact with the test FQs, resulting in a wider range of cross-reactivity values. It has been reported that the equilibrium binding constant (K_D_) of anti-nor 155 with norfloxacin (1.996 × 10^−9^) was lower than that of anti-nor 132 (1.152 × 10^−8^)^34^. A low K_D_ value indicates a high affinity between the analyte and the ligand. These findings supported the suggestion that the bindings between the FQs and anti-nor 155 had a higher affinity than those with anti-nor 132.

## Methods

### Antibody production and purification

Anti-nor 132- and anti-nor 155-producing monoclones were obtained from the Institute of Biotechnology and Genetic Engineering, Chulalongkorn University, Thailand. Monoclones had been produced by a conventional cell hybridoma preparation method^35,36^. Briefly, mice were immunized at two-week intervals with a conjugate of norfloxacin-bovine serum albumin. Splenocytes of the immunized mouse were fused with myeloma cells to generate hybridoma cells which were screened for antibody-producing hybridomas by both indirect ELISA and indirect competitive ELISA. Monoclone of the selected hybridomas were obtained by a limiting dilution culture technique. To produce mAb, cells were cultured in RPMI 1640 medium supplemented with 20% fetal calf serum at 37 °C in a 5% CO_2_ incubator. The culture medium was centrifuged at 1500 rpm for 5 min, and the antibody in the supernatant was purified using ÄKTA affinity chromatography with HiTrap Protein G HP antibody purification columns (GE Healthcare, IL). The column was equilibrated with 2 mM phosphate buffer (pH 7.0) at a flow rate of 1.0 mL/min. Unbound proteins were washed out from the column with 30 mL equilibrated buffer, and the antibody was fractionally eluted (1 mL/fraction) with 0.1 M glycine–HCl buffer (pH 2.7) into 70 µL of 1 M Tris–HCl buffer (pH 9.0). Fractions containing a high antibody concentration were combined and dialyzed against 0.01 M phosphate-buffered saline (PBS), pH 7.4 to remove salt and low molecular weight impurities. The antibodies were kept at − 20 °C until further use.

### Conjugation of FQ and ovalbumin

Norfloxacin, enrofloxacin, ciprofloxacin, and ofloxacin (Sigma-Aldrich, USA) were separately conjugated to ovalbumin using a carbodiimide active ester method modified from Watanabe et al.^37^ Briefly, drug (20 mg) N-hydroxysuccinimide (10 mg) (Sigma-Aldrich, USA) (10 mg) and 1-ethyl-3-(3-dimethylaminopropy) carbodiimide (Sigma-Aldrich, USA (10 mg) were dissolved in 1 mL of dimethylformamide (Merck, Germany). The mixture was stirred for 30 min at room temperature (25–27 °C). Then, the mixture was added dropwise into the ovalbumin solution (50 mg in 3 mL of 0.01 M phosphate-buffered saline (PBS), pH 7.4). The solution was stirred at room temperature for 2 h and then dialyzed against PBS three times. The conjugate solution was filtered through 0.2-µm cellulose acetate membrane, and the conjugate was kept at − 20 °C until use.

### Antigen-captured indirect competitive ELISA

An antigen-captured indirect competitive ELISA was used for evaluating the binding ability of the antibodies produced. Ninety-six-well plates were coated with norfloxacin–ovalbumin conjugate at 4 °C overnight. After washing for three times with washing buffer or PBST (10 mM PBS, pH 7.4 containing 0.05% Tween® 20), plates were blocked with skim milk (300 µL/well) at 37 °C for 1 h, followed by another washing step. Then, anti-nor (100 µL/well) and each FQ of interest or competitor at various concentrations (50 µL/well) were added, and the plates were incubated at 37 °C for 2 h. After three washing steps, goat anti-mouse IgG-horse radish peroxidase (Jackson Immuno, USA) was added (1:10,000 in PBS, 100 µL/well) into each well, and the plates were incubated at 37 °C for 1 h. After another three washing steps, a tetramethylbenzidine substrate solution was added (100 µL/well), and the reaction was allowed to occur for 15 min in the dark at room temperature. The enzymatic reaction was stopped by adding 1 N H_2_SO_4_ (100 µL/well), and the absorbance was measured at 450 nm using a microplate reader (Titertek multiskan model: MCC/340, Finland) was used in the experiment.

Detection sensitivity was quantified in terms of LOD and IC_50_. The LOD value was defined as the norfloxacin concentration corresponding to the point at which the mean maximum absorbance value when no competitor is present in the assay (B_0_) was decreased by three times its standard deviation. The IC_50_ value was defined as the concentration of free FQs that resulted in a 50% reduction of the B/B_0_ ratio, in which B is the absorbance value obtained from indirect competitive ELISA at different concentrations of the FQs^38^.

The specificity of each antibody was evaluated in terms of its cross-reactivity, which was calculated using the IC_50_ of norfloxacin: IC_50_ of the competitors ratio^39^ as follows:

$$\% Cross{\text{-}}reactivity = 100 \times \frac{{IC_{50} \;of\;norfloxacin}}{{IC_{50} \;of\;competitor}}.$$

### Generation of three-dimensional structures of mAbs

The amino acid sequences of the anti-nor 132 and anti-nor 155 were retrieved from GenBank. The obtained sequences were submitted to the SWISS-MODEL Automated Protein Modeling Server. The molecular structure template having highest sequence identities of VH and VL of the mAbs was chosen from the Protein Data Bank (www.pdb.org). The percentage identity between the amino acid sequences of the VH and VL regions of the mAbs and the template were aligned using the CLUSTALW server. To generate a 3D Fab fragment of the mAbs, VH and VL were superimposed with the crystal structure template of 5eoq^40^ using PyMOL software (licensed version 2.5.1, Schrödinger Inc, USA).

### Docking of antigens to the mAbs

The 3D structures of the ligands norfloxacin (CID: 4539), enrofloxacin (CID: 71188), ciprofloxacin (CID: 2764), and ofloxacin (CID: 4583) were obtained from the PubChem web site. Docking simulations of the mAbs with the ligands were performed using the molecular docking and visual screening program AutoDock Vina^41^. The AutoDock Vina could determine the docking position of the mAbs by setting the X, Y, Z dimensions of the docking grid box, which covered the whole molecule of the mAbs. The size of the grid box was set to 47.25 Å × 47.25 Å × 47.25 Å (x, y and z) with 0.375 Å spacing between the grid points. During the blind docking process, the 20 conformers of mAb-Ag complex were generated. The docking process was achieved using the command prompt into Windows 10. After the docking process finished, Vina scores showed as free binding energies were obtained from the docking calculation. The best fitted models was used to obtain the lowest free energies. Finally, the binding models were examined by PyMOL Stereo 3D Quad-buffer (licensed version 2.5.1, Schrödinger Inc, USA) and the Discovery Studio 2019 Client (free version, BIOVIA Inc, China) was used to predict the amino acid residues interacting with each FQs.

## Data Availability

All data are available upon request from the authors.
